# Comparison of Perinatal Outcomes of Letrozole-Induced Ovulation and Hormone Replacement Therapy Protocols in Patients With Abnormal Ovulation Undergoing Frozen-Thawed Embryo Transfer: A Propensity Score Matching Analysis

**DOI:** 10.3389/fendo.2022.837731

**Published:** 2022-03-16

**Authors:** Wenjuan Zhang, Zhaozhao Liu, Junwei Zhang, Bingnan Ren, Manman Liu, Jiaheng Li, Wen Zhang, Yichun Guan

**Affiliations:** Reproduction Center, The Third Affiliated Hospital of ZhengZhou University, ZhengZhou, China

**Keywords:** frozen-thawed embryo transfer, pregnancy outcomes, perinatal outcomes, letrozole-induced ovulation protocol, hormone replacement therapy protocol

## Abstract

**Background:**

With the increasing use of frozen embryo transfer (FET), the best endometrial preparation protocol is continuously being discussed. The hormone replacement therapy (HRT) cycle and letrozole-induced ovulation (L-OI) cycle are available protocols for patients with abnormal ovulation. Previous comparisons of the two protocols have focused on pregnancy outcomes, with less attention to perinatal outcomes, and population heterogeneity was large; thus, convincing conclusions about which protocol is more appropriate could not be drawn.

**Methods:**

We performed a retrospective cohort study using propensity score matching (PSM) analysis for a population of patients undergoing FET cycles in the reproductive center of the Third Affiliated Hospital of Zhengzhou University from January 2016 to September 2020. The main outcome measures were clinical pregnancy rate, live birth rate, very preterm delivery (VPTD), preterm delivery (PTD), low birth weight (LBW), macrosomia, small for gestational age (SGA), large for gestational age (LGA), hypertensive disorders of pregnancy (HDP), gestational diabetes mellitus (GDM), premature rupture of membranes (PROM), placenta previa, and congenital abnormality.

**Results:**

A total of 8010 women were enrolled. Due to the large heterogeneity among the patients, we conducted 1:1 PSM, and 1461 women matched in each group. Compared with the HRT group, the L-OI group had a smaller proportion of thin endometrium (27.38% vs. 41.07%) and thicker endometrium on the day of embryo transfer (9.63 ± 1.82 vs. 8.91 ± 1.38). There were no significant differences in clinical pregnancy rate, early abortion rate or live birth rate between the groups. There was no significant difference in perinatal outcomes of singleton live birth, including VPTD, PTD, postterm delivery, LBW, macrosomia, SGA, LGA, GDM, HDP, placenta previa, and congenital malformation.

**Conclusion:**

For women with abnormal ovulation, the pregnancy and perinatal outcomes of HRT and L-OI protocols are reassuring. It seems that both protocols are safe and effective for endometrial preparation in frozen-thawed embryo transfer in the clinic.

## Introduction

Since the first successful live birth following human frozen embryo transfer (FET) reported by Zeilmaker’s team (1), the number of FET cycles has increased steadily worldwide due to improvements in laboratory technology, especially vitrification technology, and an increase in the number of available embryos (2, 3). In addition, the “whole-embryo freezing” strategy, i.e., selective freezing of all embryos before FET, has become a suitable option, especially for patients with a high risk of ovarian hyperstimulation syndrome (OHSS), preimplantation genetic testing (PGT) and double ovarian stimulation (DuoStim), as it reduces complications while simultaneously enhancing the live birth rate (4, 5).

Recently, many studies have shown that the outcome of frozen embryo transfer cycles was not inferior to that of FET cycles (6, 7), and some studies have even suggested that FET was associated with a higher pregnancy rate and lower complication rate (8, 9). Nevertheless, a recent meta-analysis showed that FET was associated with an increased risk of hypertensive disorders of pregnancy (HDP), postterm delivery, macrosomia and large for gestational age (LGA) (10, 11), but with reduced risk of preterm birth (PTD), low birth weight (LBW) and small for gestational age (SGA) (12, 13).

Endometrial preparation protocols optimize the success rate of FET by synchronizing endometrial receptivity and embryonic development stage. Multiple protocols for endometrial preparation for FET have been explored. A natural cycle (NC), an artificial cycle with hormone replacement therapy (HRT), and a cycle with ovulation induction (OI) are the most common protocols. All three protocols are suitable for patients with normal ovulation, and the latter two are also appropriate for patients with ovulatory disorders. Several recent retrospective studies found that NC was the best choice for women with normal ovulation (14, 15); however, there is no unified conclusion on the optimal choice for patients with abnormal ovulation (16, 17).

Clomiphene (CC) and letrozole (LE) are commonly used drugs in the OI cycle. In recent years, LE has been most widely used in OI for patients with polycystic ovary syndrome (PCOS), and it is the first-line OI drug for PCOS patients (18, 19). LE, a third-generation aromatase inhibitor, is commonly used in the clinic because it does not consume estrogen receptors, maintains a normal central feedback system, and promotes normal follicular growth, and it has no negative impact on the endometrium (20, 21) or pregnancy or fetal development (22). Because of its convenience, low cost and time controllability, HRT cycles have been widely applied for patients with abnormal ovulation (2, 23).

Recent studies have demonstrated that by using exogenous estrogen and progesterone to prepare the endometrium and inhibit ovulation, the HRT protocol in FET affected maternal and neonatal outcomes, resulting in the loss of the corpus luteum (CL), which can lead to adverse perinatal outcomes (14, 24). However, at present, there are few studies on the specific population of patients with abnormal ovulation, and heterogeneity in this population is large. Furthermore, as most comparisons between HRT and OI cycles have focused on the clinical pregnancy rate or live birth rate and paid little attention to maternal and neonatal outcomes, convincing conclusions cannot be drawn.

Therefore, this study aimed to explore the relationship between exposure of patients with abnormal ovulation to different endometrial preparation protocols and pregnancy and perinatal outcomes, including pregnancy rate, live birth rate, adverse obstetric complications and neonatal outcomes, to further optimize maternal and infant health after FET in patients with abnormal ovulation.

## Materials and Methods

### Patients

A total of 8010 women who were undergoing FET cycles from January 2016 to September 2020 at our center were enrolled. We included FET cycles of oligoanovulation (menstrual cycle>37 d), anovulation with letrozole-induced ovulation (L-OI) or HRT after *in vitro* fertilization (IVF)/intracytoplasmic sperm injection (ICSI). The following exclusion criteria were applied: 1) maternal age >40 years; 2) adenomyosis, uterine malformations or recurrent miscarriage; and 3) use of donor oocytes and PGT cycles.

### Endometrial Preparation Protocols

For HRT cycles, patients were prescribed 2 mg of estradiol valerate (Bayer Co., Germany) to be taken orally three times daily, starting at days 2-4 of menstruation for 7 days. Then, the drug dose was adjusted according to the thickness of the endometrium (up to 9 mg per day). Endometrial transformation was performed when the medication was taken for more than 12 days and endometrial thickness was ≥7 mm; the cycle was cancelled if endometrial thickness was less than 7 mm.

LE (2.5 mg/5 mg) was administered orally for 5 days on the 3rd-5th days of menstruation, and the follicular development speed was monitored by ultrasound. If follicular development was poor, HMG (Lizhu Pharmaceutical Trading Co., China) (37.5-75 IU daily) was added as appropriate to aid in the development of follicles. When the dominant follicle developed to 14 mm, the serum luteinizing hormone (LH) level indicated that ovulation was about to occur, the estradiol (E2) level was more than 150 pg/mL, and the endometrium thickness was more than 7 mm, 10,000 IU urinary hCG was injected (Lizhu Pharmaceutical Trading Co., China). Endometrial transformation was then performed. The cycle was cancelled if follicular dysplasia occurred.

For HRT or L-OI cycles, oral dydrogesterone (2 times daily, 10 mg once) (Abbott Co. USA) and intravaginal administration of 90 mg of a progesterone sustained-release vaginal gel (Merck Co. Germany) were given as luteal phase support until the 12th week of pregnancy. The same dose of estrogen valerate as before transformation was taken until 14 days after embryo transfer. In the case of pregnancy, the drug was continued until clinical pregnancy, which was defined as the presence of an intrauterine gestational sac by ultrasonography at 7–8 weeks of gestation.

### Data Collection and Outcome Definition

Patient characteristics, such as age, body mass index (BMI), type of infertility, indication for IVF, duration of infertility, basal serum follicle stimulating hormone (FSH), basal antral follicle count (AFC), the number of previous FET failures, endometrial thickness, number of transferred embryos, developmental stage of embryo, pregnancy or live birth, and singleton or twins, were collected through the electronic case system of our center.

For patients with a gestational sac echo and singleton live birth after embryo transfer, pregnancy complications were collected during a telephone follow-up and recorded by a designated nurse in our center. Maternal and neonatal outcomes were recorded and classified according to the information provided by the patients.

Early spontaneous abortion was defined as a clinical pregnancy that failed to reach the 12th gestational week. Live birth was defined as the birth of a live child after 28 weeks of gestation per embryo transfer cycle. Very preterm delivery (VPTD), preterm delivery (PTD), term birth and postterm delivery were defined as a baby born after <32 weeks, <37 weeks of gestation, ≤37 weeks ≤ 41 weeks and >41 weeks of gestation, respectively. The neonatal birth weight of singleton live births was as follows: LBW (<2500 g), SGA (<10th percentile for gestational age) (25), macrosomia (≥4000 g), and LGA (>90th percentile for gestational age) (25).

### Statistical Analysis

All statistical management and analyses were performed using SPSS software, version 22.0.

Because there was obvious heterogeneity in basic characteristics, the data were analyzed after 1:1 propensity score matching (PSM).

The one-sample K-S test was used to check for normality. Continuous variables with abnormal distributions are expressed as the mean ± SD, and Student’s t test was used to assess between-group differences. Categorical variables are represented as the number of cases (n) and percentage (%).

Means from chi-square analyses were used to assess differences between the groups. Multiple logistic regression was applied to further analyze different items. Unadjusted odds ratios and adjusted odds ratios with 95% confidence intervals (CIs) were calculated. Statistical significance was set at *P*<0.05.

## Results

### Study Population

From January 2016 to September 2020, 8010 FET cycles were evaluated according to the inclusion and exclusion criteria. There were 6549 patients in the HRT group and 1461 patients in the L-OI group. We separately analyzed the patients with a gestational sac echo and singleton live birth after embryo transfer, with 395 patients in the HRT group and 457 in the L-OI group.

### Baseline Characteristics

**Figure 1:**
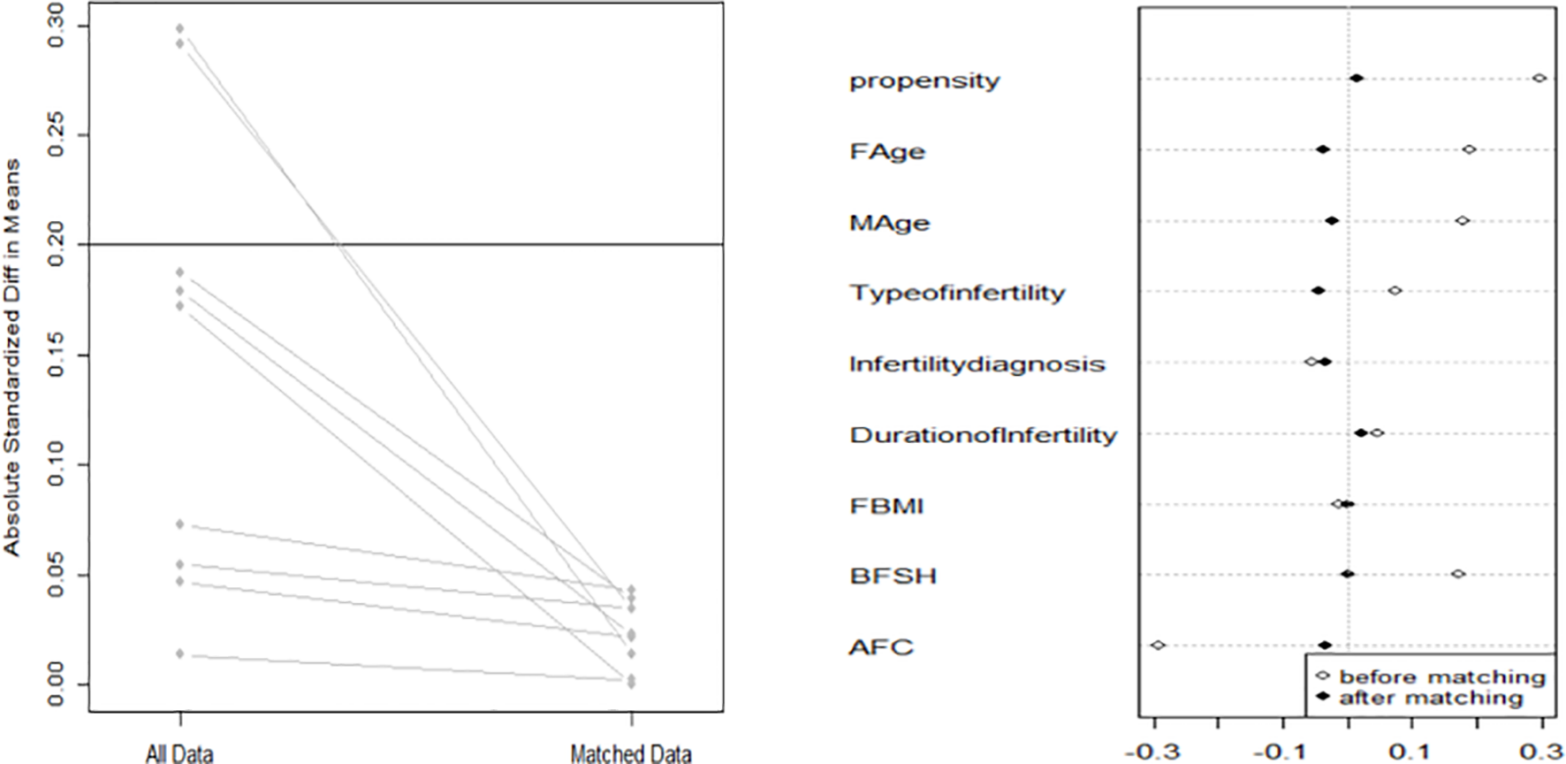
After 1:1 PSM, the data heterogeneity between the two groups was significantly reduced.

When comparing basic characteristics between the two groups, we found that there were differences in female and male age, type of infertility, indication for IVF, duration of infertility, basal serum FSH, and basal AFC (**Table 1**). Therefore, based on these differences, we conducted 1:1 PSM, and 1461 women were matched in each group. After matching, there were no significant differences in basic characteristics between the groups (**Table 2** and **Figure 1**).

**Table 1:** Patient clinical characteristics.

Characteristics	HRT (6549)	L-OI (1461)	P value
Female age (y)	31.14 ± 4.40	30.31 ± 4.06	0.000
Male age (y)	32.20 ± 5.34	31.25 ± 4.56	0.000
Body mass index (kg/m2)	23.98 ± 3.29	24.02 ± 3.16	0.634
Type of infertility			0.012
Primary infertility	42.80% (2803/6549)	46.41% (678/1461)	
Secondary infertility	57.20% (3746/6549)	53.59% (783/1461)	
Indication for IVF			
Tubal factor	36.11% (2365/6549)	31.35% (458/1461)	0.001
Endometriosis	0.37% (24/6549)	0.55% (8/1461)	0.321
Ovulatory dysfunction	12.98% (850/6549)	14.37% (210/1461)	0.155
Male factor	17.50% (1146/6549)	21.01% (308/1461)	0.001
Others	5.08% (333/6549)	5.61% (82/1461)	0.410
Mixed factors	27.96% (1831/6549)	27.04% (395/1461)	0.477
Duration of Infertility (y)	3.47 ± 2.84	3.34 ± 2.67	0.000
Basal serum FSH level (IU/L)	7.20 ± 19.05	6.15 ± 2.25	0.000
Basal antral follicle count	17.76 ± 8.15	20.14 ± 7.22	0.000

Data are presented as the mean ± SD for continuous variables and % (n/N) for categorical variables. Student’s t test was used for continuous variables, and the Pearson’s chi-squared test was used for categorical variables with Fisher’s exact test when necessary.

**Table 2:** Patient clinical characteristics after PSM.

Characteristics	HRT (1461)	L-OI (1461)	P value
Female age (y)	30.14 ± 4.04	30.31 ± 4.06	0.250
Male age (y)	31.12 ± 4.83	31.25 ± 4.56	0.479
Body mass index (kg/m2)	24.03 ± 3.38	24.02 ± 3.16	0.773
Type of infertility			0.251
Primary infertility	48.53% (709/1461)	46.41% (678/1461)	
Secondary infertility	51.47% (752/1461)	53.59% (783/1461)	
Indication for IVF			
Tubal factor	34.84% (509/1461)	31.35% (458/1461)	0.045
Endometriosis	0.41% (6/1461)	0.55% (8/1461)	0.592
Ovulatory dysfunction	12.80% (187/1461)	14.37% (210/1461)	0.214
Male factor	18.89% (276/1461)	21.08% (308/1461)	0.139
Others	4.93% (72/1461)	5.61% (82/1461)	0.408
Mixed factors	28.13% (411/1461)	27.04% (395/1461)	0.508
Duration of Infertility (y)	3.40 ± 2.61	3.34 ± 2.67	0.533
Basal serum FSH level (IU/L)	6.15 ± 2.58	6.15 ± 2.25	0.986
Basal antral follicle count	19.85 ± 7.35	20.14 ± 7.22	0.295

Data are presented as the mean ± SD for continuous variables and % (n/N) for categorical variables. Student’s t test was used for continuous variables, and the Pearson’s chi-squared test was used for categorical variables with Fisher’s exact test when necessary.

We found that the number of previous FET failures was higher in the L-OI group than that of the HRT group. In terms of clinical data, the endometrium was thicker and the proportion of thin endometrium lower in the L-OI group **(** **Table 3**).

**Table 3:** Patient clinical and embryological characteristics.

Characteristics	HRT (1461)	L-OI (1461)	P value
Number of previous FET failures	0.11 ± 0.330	0.16 ± 0.422	0.000
Endometrial thickness on the day of embryo transfer (mm)	8.91 ± 1.38	9.63 ± 1.82	0.000
Thin endometrium	41.07% (60/1461)	27.38% (40/1461)	0.042
Number of transferred embryos	1.47 ± 0.50	1.42 ± 0.49	0.002
One	52.57% (768/1461)	58.38% (853/1461)	
Two	47.43% (693/1461)	41.62% (608/1461)	
Development stage of the embryo			0.604
D3	31.69% (463/1461)	30.80% (450/1461)	
D5/D6	68.31% (998/1461)	69.20% (1011/1461)	

Data are presented as the mean ± SD for continuous variables and % (n/N) for categorical variables. Student’s t test was used for continuous variables, and the Pearson’s chi-squared test was used for categorical variables with Fisher’s exact test when necessary.

FET, Frozen embryo transfer.

### Clinical Outcomes

In terms of clinical outcome, there were no significant differences in clinical pregnancy rate, early abortion rate or live birth rate between the two groups, but the twin rate was higher in the HRT group, which may be because the number of transferred embryos was greater than that in the L-OI group **(** **Table 4**).

**Table 4:** Clinical outcomes.

	HRT (1461)	L-OI (1461)	P value
Clinical pregnancy rate	50.17% (733/1461)	49.01% (716/1461)	0.529
Early spontaneous abortion rate	10.95% (160/1461)	9.38% (137/1461)	0.159
Live birth rate	36.41% (532/1461)	37.99% (555/1461)	0.379
Singletons	78.01% (415/532)	85.05% (472/555)	0.003
Twin	21.99% (117/532)	14.95% (83/555)	

Data are presented as % (n/N) for categorical variables. The Pearsonc2 test was used for categorical variables with Fisher’s exact test when necessary.

Regarding the main outcome measures, we conducted a multiple logistic regression analysis to adjust for the influence of confounding factors. The included factors were female age, number of previous FET failures, BMI, AFC, endometrial thickness on the day of embryo transfer, thin endometrium and number of transferred embryos. After adjustments for confounding factors, the clinical pregnancy rate, early spontaneous abortion rate, live birth rate and twin rate were not significantly different between the groups **(** **Table 5** **)**.

**Table 5:** Unadjusted and adjusted odds ratios of pregnancy outcomes following L-OI versus HRT cycles.

	Unadjusted OR (95%CI)	Adjusted OR (95%CI)
Clinical pregnancy rate	1.048 (0.906-1.211)	1.086 (0.93-1.267)
Early spontaneous abortion rate	1.189 (0.934-1.512)	1.248 (0.973-1.602)
Live birth rate	0.932 (0.802-1.083)	0.951 (0.808-1.120)
Twins	1.606 (1.177-2.191)	1.431 (0.994-2.060)

The analysis was adjusted for female age, number of previous FET failures, BMI, AFC, endometrial thickness on the day of embryo transfer, thin endometrium and number of transferred embryos. CI, Confidence interval; FET, Frozen-thawed embryo transfer.

We mainly analyzed maternal and neonatal outcomes and observed no significant differences in perinatal outcomes, including VPTD, PTD, postterm delivery, LBW, macrosomia, SGA, LGA, GDM, HDP, placenta previa, and congenital malformation, between the groups (**Table 6** **)**. The same conclusion was reached after further multiple logistic regression analysis (**Table 7**).

**Table 6:** Perinatal and neonatal outcomes of singleton live birth.

	HRT (395)	L-OI (457)	P value
VPTD	0.25% (1/395)	0.66% (3/457)	0.390
PTD	5.82% (23/395)	6.13% (28/457)	0.852
Term birth	86.33% (341/395)	87.96% (402/457)	0.476
Postterm delivery	7.59% (30/395)	5.25% (24/457)	0.162
Neonatal weight (g)	3459.86 ± 468.92	3419.74 ± 519.33	0.240
Newborn’s sex			0.441
Male	54.94% (217/395)	52.30% (239/457)	
Female	45.06% (178/395)	47.70% (218/457)	
LBW	2.78% (11/395)	3.06% (14/457)	0.810
Macrosomia	10.38% (41/395)	10.72% (49/457)	0.871
SGA	5.06% (20/395)	5.69% (26/457)	0.687
LGA	18.23% (72/395)	17.72% (81/457)	0.849
GDM	7.85% (31/395)	8.32% (38/457)	0.803
HDP	6.33% (25/395)	4.60% (21/457)	0.264
PROM	5.06% (20/395)	3.58% (17/457)	0.337
Placenta previa	0.25% (1/395)	0.44% (2/457)	0.555
Others	0.76% (3/395)	1.09% (5/457)	0.882
Congenital malformation	1.27% (5/395)	0.22% (1/457)	0.158

Data are presented as the mean ± SD for continuous variables and % (n/N) for categorical variables. Student’s t test was used for continuous variables, and the Pearson’s chi-squared test was used for categorical variables with Fisher’s exact test when necessary.

VPTD, very preterm delivery; PTD, preterm delivery; LBW, low birth weight; SGA, small for gestational age; LGA, large for gestational age; GDM, gestational diabetes mellitus; HDP, hypertensive disorders of pregnancy; PROM, premature rupture of membranes.

**Table 7:** Unadjusted and adjusted odds ratios of perinatal and neonatal outcomes of singleton live birth undergoing L-OI versus HRT FET cycles.

	Unadjusted OR (95%CI)	Adjusted OR (95%CI)
Very preterm delivery (VPTD)	0.384 (0.040-3.707)	0.222 (0.021-2.368)
Preterm delivery (PTD)	0.947 (0.536-1.637)	1.054 (0.585-1.898)
Postterm delivery	1.483 (0.852-2.582)	1.147 (0.643-2.049)
LBW	0.906 (0.407-2.020)	0.875 (0.385-1.989)
Macrosomia	0.964 (0.622-1.496)	0.951 (0.607-1.491)
SGA	0.687 (0.884-1.610)	0.751 (0.404-1.398)
LGA	0.849 (0.729-1.469)	1.122 (0.780-1.614)
GDM	0.925 (0.573-1.494)	0.959 (0.584-1.575)
HDP	1.384 (0.780-2.456)	1.218 (0.678-2.187)
Placenta previa	0.577 (0.052-6.392)	0.881 (0.067-11.634)
Congenital malformation	5.846 (0.680-50.253)	5.370 (0.606-47.591)

The analysis was adjusted for female age, number of previous FET failures, BMI, AFC, endometrial thickness on the day of embryo transfer, thin endometrium and number of transferred embryos.

CI, confidence interval; FET, frozen embryo transfer; VPTD, very preterm delivery; PTD, preterm delivery; LBW, low birth weight; SGA, small for gestational age; LGA, large for gestational age; GDM, gestational diabetes mellitus; HDP, hypertensive disorders of pregnancy; PROM, premature rupture of membranes.

## Discussion

Our study showed no difference in pregnancy rate, live birth rate or abortion rate between HRT and L-OI cycles for patients with abnormal ovulation. Moreover, there was no difference between the two groups regarding perinatal outcomes.

For patients with abnormal ovulation, both HRT and L-OI are common endometrial preparation protocols in the clinic. The safety and OI effect of LE have also been generally recognized (26). Previous studies have suggested that L-OI cycles resulted in a higher live birth rate than HRT cycles; nevertheless, these studies did not examine maternal and neonatal outcomes (16, 27). Interestingly, some studies reached the same conclusions as in our study. A prospective study including 116 PCOS patients reported similar clinical pregnancy rates for HRT and L-OI protocols (28). Another randomized controlled study including 100 patients found that the L-OI protocol did not improve pregnancy outcomes compared with HRT (17). However, these studies did not focus on maternal and infant outcomes.

Despite no difference in pregnancy outcomes between the two groups, endometrial thickness on the day of embryo transfer in the L-OI group was greater than that in the HRT group in our study, which was consistent with a previous study from our center (29). The reason may be that the proportion of patients with thin endometrium was relatively high in the HRT group. In addition, LE has no negative effects on endometrial or cervical mucus. LE can enable full endometrial pinopode expression and increase integrin αvβ3 expression in the endometrium during implantation (18, 30). LE further decreases intraovarian and serum estrogen levels by blocking conversion of androgens to estrogens in ovarian granulosa cells (20). Subsequently, low estrogen levels reduce ubiquitination of estrogen receptors. This process leads to faster endometrial proliferation and increased blood levels in the uterus and endometrium, with positive effects on pregnancy outcomes (31, 32). Thus, L-OI can be used to prepare endometrial tissue for FET for patients with thin endometrial tissue. However, a previous study in our center reported that L-OI cycles were associated with a higher live birth rate than HRT cycles. Although the live birth rate was increased in our study by using the L-OI protocol, there was no significant differences between the groups possibly due to a large difference in the number of cases included in the L-OI (502) and HRT (2280) groups. There was also heterogeneity in basic characteristics; a previous study adopted regression analysis for correction (29), which was different from the 1:1 PSM in our study.

Moreover, our study found no significant difference in maternal or infant health between the two groups. A large retrospective cohort study in Japan in 2017 that included 110,772 FET cycles, which were divided into an LE-induced ovulation group, NC group and HRT group according to the endometrial preparation protocol used, found that neonatal outcomes of the different treatment schemes were basically similar, consistent with our results. A previous study in our center also reached similar conclusions (29).

There have been few studies on the perinatal complications and infant safety of the two protocols. Studies have shown that newborns were likely to have LBW and macrosomia after HRT cycles (14, 33) while pregnant women had an increased risk of HDP and cesarean section (15, 34). Saito et al.’s study suggested that the HRT cycles were associated with a higher risk of HDP and placental implantation and a lower risk of GDM (35). Another meta-analysis demonstrated that compared with the NC protocol, the OI protocol was associated with an increased incidence of PTD and LBW (36). However, the studies mentioned above compared three protocols, and the study population was not limited.

Recent studies have shown that the HRT protocol lacks CL, which is a crucial hormone for embryo implantation, placenta and pregnancy maintenance. Recent studies emphasized that loss of CL is associated with altered vascular health and insufficient cardiovascular adaptation in early pregnancy, leading to the occurrence of preeclampsia, affecting placental formation and causing placental hyperplasia (37, 38), with impacts on the mother and newborn. CL not only provides estrogen and progesterone but also vasoactive substances, such as relaxin and vascular endothelial growth factor, which may be important for placental formation. These substances are not available in the HRT cycle, which may increase the incidence of obstetric complications (39, 40). In our study, there was no difference between the two groups with regard to singleton delivery. The reason may be due to the different doses and types of luteal support after FET.

In FET cycles, it is necessary to add progesterone to obtain sufficient corpus luteum support to obtain a good pregnancy outcome due to the lack of endogenous progesterone production. In our study, corpus luteum support was provided by a combination of oral and vaginal administration, and the dose was sufficient. In Hu et al. ’s study, only oral dydrogesterone (20 mg/d) was applied as luteal support in HRT cycles (14). Previous studies have suggested that dydrogesterone alone was likely not effective as a monotherapy in FET (41) but that the combination of oral and vaginal administration increased the concentration of progesterone in the serum and endometrium and improved the reproductive outcome (42, 43). In the study of Zong et al. dydrogesterone (40 mg/d) and progesterone capsules (Utrogestan, Capsugel) (200 mg/d) were given as luteal-phase support in HRT and OI cycles (33). This was not consistent with our study, in which oral dydrogesterone (60 mg/d) and intravaginal administration of 90 mg of a progesterone sustained-release vaginal gel were given as luteal-phase support. A recent meta-analysis suggested that once-daily Crinone gel or micronized progesterone (200 mg) three times per day is the most suitable luteal support dose (44).

Another reason may be that in some studies, when there were significant differences in basic characteristics and obvious differences in the number of included populations between the groups, logistic regression was used to correct confounding factors instead of ex ante PSM. Although they could correct some confounding factors, the statistical effectiveness did not seem to be more convincing than PSM.

Several limitations associated with this study warrant mentioning. 1) The number of samples was lower after PSM than before, and the study was a retrospective study with some deviation; hence, additional prospective research is needed to verify our results. 2) Patients with diabetes and hypertension were not excluded, but blood pressure and blood glucose were controlled normally before FET, which might have led to some inaccuracy in the results. 3) Because maternal complications and offspring outcomes were obtained by telephone and reported by patients, incomplete and missing data were present. 3) Not all the patients included were undergoing their first FET cycle, though the number of previous transplantation failures between the two groups was compared, some bias in outcome may exist.

In conclusion, for women with abnormal ovulation undergoing FET, both HRT and L-OI protocols are safe and effective in the clinic. Although maternal and infant outcomes appear to be reassuring, they need to be confirmed by additional prospective research with large samples.

## Data Availability Statement

The raw data supporting the conclusions of this article will be made available by the authors, without undue reservation.

## Ethics Statement

The studies involving human participants were reviewed and approved by the Ethics Committee of the Third Affiliated Hospital of Zhengzhou University. Written informed consent for participation was not required for this study in accordance with the national legislation and the institutional requirements.

## Author Contributions

WZ and YG designed the study and selected the population to be included and excluded. WZ and ZL were involved in the data extraction and analysis. JZ and BR reviewed the data. WZ and ZL were involved in drafting this article. ML, JL, and WZ modified the manuscript. All authors contributed to the article and approved the submitted version.

## Conflict of Interest

The authors declare that the research was conducted in the absence of any commercial or financial relationships that could be construed as a potential conflict of interest.

## Publisher’s Note

All claims expressed in this article are solely those of the authors and do not necessarily represent those of their affiliated organizations, or those of the publisher, the editors and the reviewers. Any product that may be evaluated in this article, or claim that may be made by its manufacturer, is not guaranteed or endorsed by the publisher.
